# Moderated Mediating Mechanism Effects of Chinese University Entrepreneurship Education on Independent Student Entrepreneurship

**DOI:** 10.3389/fpsyg.2022.782386

**Published:** 2022-02-14

**Authors:** Yuhui Li, Yimin Sha, Yijun Lv, Yenchun Jim Wu, Haiming Liu

**Affiliations:** ^1^School of Innovation and Entrepreneurship, Wenzhou Medical University, Zhejiang, China; ^2^College of Humanities and Arts, National Taipei University of Education, Taipei, Taiwan; ^3^Graduate Institute of Global Business and Strategy, National Taiwan Normal University, Taipei, Taiwan; ^4^School of Entrepreneurship, Wenzhou Polytechnic, Zhejiang, China

**Keywords:** moderated mediating mechanism, independent entrepreneurship, entrepreneurship education, entrepreneurship opportunity identification, entrepreneurial experiences

## Abstract

Entrepreneurship education plays a mediating moderating role in independent entrepreneurship, especially for the fresh graduates where entrepreneurial knowledge charms. Based on the mediating effect model, this study explores the correlation between three factors and independent entrepreneurship. A set of hypotheses was established by investigating the theoretical background within the field of interest. Such hypotheses were later assessed by an online-offline mix study conducted among graduates. The result found that entrepreneurship theory-based courses can promote independent entrepreneurship, but entrepreneurship practice training surprisingly failed to promote. Entrepreneurial opportunity identification mediated only between theory-based courses and independent entrepreneurship. The findings found could be highly beneficial in organizing entrepreneurship syllabus, scientifically arranging a theory-based course, and practicing a training course. Moreover, it could be further developed into a pedagogical model.

## Introduction

Our generation is currently confronting huge challenges such as disease controls, poverty threatens, and economic unsustainability. The promotion of independent entrepreneurship can play a vital role in addressing these challenges (Stephan et al., 2016). Since 2015, the Chinese government has successively issued a number of documents and then launched work plans related to the reform of entrepreneurship education in the universities, aiming at gradually improving the entrepreneurship assistance policy. Meanwhile, the entrepreneurship education system has been reformed, attempting to transfer its main character from academic training to cultivated independent entrepreneurs. According to the *Employment Report of Chinese College Students* (*Employment Blue Book*), Chinese university students who have chosen to start their own businesses during a postgraduation period of 3 years have risen from 5.7% in 2015 to 8.1% in 2019. Achievements have been made, whereas the self-employment rate of Chinese university students is not optimistic, and independent entrepreneurship right after graduation has dropped from 3.0% in 2015 to 2.7% in 2019 (Mycos Research Institute, 2016, 2017, 2018, 2019, 2020). Students were able to achieve a better understanding of a panorama with decisions that were not a decent match for their personalities or goals in life. It would be a beneficial effect despite the drop in entrepreneurial activity.

Regarding independent entrepreneurship, scholars have explored the possibility to reveal a certain relationship between entrepreneurship factors and independent entrepreneurship. The studies have focused on entrepreneurship education (Huang et al., 2020), entrepreneurship policy (Graevenitz et al., 2010), and entrepreneurship culture. The entrepreneurship education featured the least flexibility to meet the requirement of cultivating a successful independent entrepreneur due to the technical, administrative, and legal problems in entrepreneurship education reform at the national level. However, based on the logical relationship between entrepreneurial education and entrepreneurial opportunity identification, it is rare to explore the moderating mechanism of entrepreneurial education on independent entrepreneurship. This study analyzes the mechanism of entrepreneurial education on independent entrepreneurship from the mediating effect of entrepreneurial opportunity identification and the moderating mechanism of entrepreneurial experience between entrepreneurial opportunity identification and independent entrepreneurship. The current pandemic has had a wide range of negative impacts on public health, social structure, and economic activities. The 2020/2021 GEM Global Report (Global Entrepreneurship Monitor, 2021) has pointed out that entrepreneurial intention is urgently needed for younger entrepreneurs. However, the digital featured new entrepreneurial activities are also more likely to encourage young entrepreneurs (Radović-Marković et al., 2009). The studies usually span at least 3 years, in both government official release and academic paper published. But the global situation requires more timely entrepreneurial studies to release the key to global economic recovery, just as vaccination is the key to global health recovery. The sampling in this study has covered students who have received entrepreneurship education within 1 year after graduation, as it has tried to present entrepreneurial details among those being actively impacted by entrepreneurship education. Such effect will decay over time after graduation, and then the 1-year window was chosen in the cross-sectional study design.

## Literature Review

### Independent Entrepreneurship

Shane and Venkataraman (2000) have defined entrepreneurship as a process that includes the discovery, identification, measurement, and utilization of entrepreneurial opportunities. It is the result of the joint action of individual endogenous and external supporting factors (Lim et al., 2016). Independent entrepreneurship, as independently exploits the contents of entrepreneurship (Margolis, 2014), is widely used in entrepreneurship-related research (Clark and Drinkwater, 2010; Kwon et al., 2013; Falco and Haywood, 2016). For example, Ajzen (1991) has constructed a model of the correlation between entrepreneurial behavior and entrepreneurial willingness based on the theory of planned behavior. The model considers that the emergence of entrepreneurial behavior is influenced by the factors of individuals, such as ability and attitude, and external environment, such as social norms. In terms of internal factors, entrepreneurs identify new entrepreneurial opportunities based on their own knowledge accumulation and practical experience and then decide whether to conduct independent entrepreneurship (Zampetakis et al., 2017). For external factors, the implementation of entrepreneurial behavior is influenced by the entrepreneurial experience of family members (Giannetti and Simonov, 2009), the level of educational institutions (Hrsman and Daghbashyan, 2014), and the overall entrepreneurial atmosphere of society (Shirokova et al., 2016). In addition, Fayolle et al. (2006) believed that entrepreneurship education would affect the behavior of independent entrepreneurship of college students. For instance, entrepreneurship education can improve entrepreneurial willingness. Students specialized in science and engineering have better entrepreneurial abilities after receiving entrepreneurship education (Liu et al., 2019). The influence of different types of entrepreneurship education on entrepreneurship behavior implementation is also different. For example, there are significant differences in the tendency of choosing independent entrepreneurship between the students receiving entrepreneurship theory education and those who receive entrepreneurship practice training (Panagiotis and Dimo, 2014).

### Entrepreneurship Education

Entrepreneurship education refers to the theory that teachers pass entrepreneurial knowledge and skills to the students (Gorman et al., 1997) and practice training in the hands-on activities (Rideout and Gray, 2013). The impact of entrepreneurial education was and still is everywhere, from an entity such as enterprises (Dana et al., 2021) to an abstract concept such as economic transition (Rachwal et al., 2016). Theory-based entrepreneurship education plays a fundamental role in entrepreneurship education (Fiet, 2001; Salamzadeh et al., 2014; Gorostiaga et al., 2019), which is helpful to improve the rate of independent entrepreneurship of college students (Zhao et al., 2005; Vukmirović, 2019), entrepreneurial ability (Detienne and Chandler, 2004), and entrepreneurial willingness (Wannamakok and Liang, 2019; Botha, 2020). Entrepreneurial practice can avoid the disconnection between entrepreneurial knowledge and entrepreneurial practice (Salamzadeh et al., 2013; An and Xu, 2021). Besides, entrepreneurial practice has a significant impact on the improvement of entrepreneurial ability (Morris et al., 2013). Both types of education can be regarded as taking a class in person or online (Radović-Marković et al., 2009), while the practice training features various formalities such as street vending, entrepreneurial contest, and internship. Durations for theory-based courses and practice-based courses are not strictly stipulated, but theory-based courses last generally longer for most Chinese universities. The ultimate purpose of a successful course design for college entrepreneurship education should be at satisfying industrial expectations (Wu and Chen, 2019).

### Entrepreneurial Opportunity Identification

There has been an important and long-term debate over entrepreneurial opportunities whether they should be recognized as being objective or subjective. The discovery views believed that opportunity is an objective existence that needs to be discovered (Hayek, 1937; Casson, 1982; Krueger, 2000; Shane and Venkataraman, 2000). The creation views suggested that entrepreneurial opportunity was a subjective procedure generated in the entrepreneurial interaction (Schumpeter, 1934, 1942; Alvarez and Barney, 2013). Despite the disagreement between the discovery views and creation views, a consensus has been reached to define entrepreneurial opportunities, i.e., the phrasing may vary, and entrepreneurial opportunities should be competitive situations in which goods and services can be introduced and sold at greater than their cost of production (McBride and Wuebker, 2021). Under the perspective of the creation viewpoint, every opportunity was supposed to be constructed, which was spontaneously exploitable to the entrepreneur, regardless of the identification procedure. Thus, entrepreneurial opportunity identification could only be linked to the discovery or recognition. The core of entrepreneurial opportunity identification was associated with information processing that was shaped by personally relevant factors such as knowledge acquired, growth environment, and innovation sparks (Vaghely and Julien, 2010). When opportunity got perceived, the ability of entrepreneurial opportunity identification directly affects the result of entrepreneurship (Krueger, 2000). To a certain extent, they affect the achievement of entrepreneurship goals or entrepreneurial benefits (Garud and Giuliani, 2013).

### Entrepreneurial Experience

Entrepreneurial experience refers to the entrepreneurial activities carried out by entrepreneurs before the establishment of a new enterprise (Stuart and Robert, 1990). It not only helps individuals to acquire entrepreneurial knowledge and skills from past experience (Tornikoski and Newbert, 2007), as well as master valuable market and product information (Clarysse et al., 2013), but also helps entrepreneurs to identify entrepreneurial opportunities in related fields (Yu et al., 2021). Entrepreneurial experience can affect the behavior of entrepreneurs (Tihula and Huovinen, 2010), for example, it can affect the individual judgment on the feasibility of entrepreneurial opportunities (Canavati et al., 2021), give full play to the ability of entrepreneurial opportunity identification (Ucbasaran et al., 2003), and improve entrepreneurial willingness (Graevenitz et al., 2010).

## Research Hypothesis

For university students with entrepreneurial intentions, entrepreneurship education can provide them with the knowledge to carry out their own entrepreneurial activities (Wilson et al., 2007). It also has a positive impact on the choice of independent entrepreneurship. Entrepreneurship curricular teaching plays a positive role in developing entrepreneurial willingness (Fayolle and Gailly, 2013) and entrepreneurial ability of students (Jones et al., 2017). Entrepreneurial practice activities help to improve the entrepreneurial ability and willingness of college students, guiding them to start their own businesses (Fiore et al., 2019). Therefore, the following hypotheses were proposed:

**H1a**: Entrepreneurship theory-based course is positively related to independent entrepreneurship.

**H1b**: Entrepreneurship practice training is positively related to independent entrepreneurship.

Entrepreneurial opportunity identification can be cultivated through entrepreneurial education (Itelvino et al., 2018). It is discovered that entrepreneurial courses and entrepreneurial practice training have positive effects on the cultivation of entrepreneurial opportunity identification ability. For example, based on a Solomon-Four-Group-Designed experiment, entrepreneurship theory-based courses have a positive effect on entrepreneurial opportunity identification (Detienne and Chandler, 2004). Entrepreneurship practice training will help students to grasp opportunities in the entrepreneurial environment full of risks and uncertainties (Neck and Greene, 2011). Therefore, the following hypotheses were proposed:

**H2a**: Entrepreneurship theory-based course is positively related to entrepreneurial opportunity identification.

**H2b**: Entrepreneurial practice training is positively related to entrepreneurial opportunity identification.

As a prerequisite for the implementation of entrepreneurial behavior, entrepreneurial opportunity identification has a positive impact on the choice of individuals to start their own business (Alvarez et al., 2013), especially for college students. Fitzsimmons and Douglas (2011) performed an analysis of 414 MBA students from Australia, China, India, and Thailand, and it was transpired that the higher the entrepreneurial opportunity identification ability can lead to stronger independent entrepreneurship tendency. Therefore, the following hypothesis was proposed.

**H3**: Entrepreneurial opportunity identification is positively related to independent entrepreneurship.

After receiving entrepreneurship education, the entrepreneurial opportunity identification ability of college students has been improved (Itelvino et al., 2018). This ability helps college students to identify and use entrepreneurial opportunities in the market to carry out independent entrepreneurship. Therefore, entrepreneurial opportunity identification plays a mediating role between entrepreneurship education and independent entrepreneurship (Gielnik et al., 2013). In addition, after statistical analysis of the questionnaire data of 291 college students from six universities in Palestine, it was found that entrepreneurial opportunity identification had a mediating function between entrepreneurship education and the choice of independent entrepreneurship (Nidal and Norashidah, 2017). Combined with hypotheses **H2a**, **H2b,** and **H3**, when entrepreneurship theory-based course and entrepreneurship practice training were set as antecedents, entrepreneurial opportunity identification had a mediating effect between them and independent entrepreneurship. Therefore, the following hypotheses were proposed:

**H4a**: Entrepreneurial opportunity identification plays a mediating role between entrepreneurial theory-based courses and independent entrepreneurship.

**H4b**: Entrepreneurial opportunity identification plays a mediating role between entrepreneurial practice training and independent entrepreneurship.

Compared with those without entrepreneurial experience, entrepreneurs with entrepreneurial experience have a higher identification level of entrepreneurial opportunities and are more likely to seize valuable entrepreneurial opportunities (Ucbasaran et al., 2003). In addition, entrepreneurs with entrepreneurial experience can also identify the authenticity of entrepreneurial opportunities through their experience (Baldacchino, 2013). In view of the improvement of entrepreneurial opportunity identification level, college students with entrepreneurial experience will better understand entrepreneurship and then choose to start their own business (Graevenitz et al., 2010). Therefore, the following hypothesis was proposed:

**H5**: Entrepreneurial experience plays a positive moderating role between entrepreneurial opportunity identification and independent entrepreneurship.

Taking hypotheses **H4a**, **H4b,** and **H5** into consideration, it was recognized that college students holding entrepreneurial experience could have a higher level of entrepreneurial opportunity identification ability, in comparison with those who have never attached such entrepreneurial experience. Meanwhile, based on the accumulation of knowledge in the process of entrepreneurship education, college students with entrepreneurial experience could have advantages in terms of applying theoretical knowledge to practice and seizing entrepreneurial opportunities from practice, namely the implementation of independent entrepreneurship action. Therefore, the following hypotheses were proposed:

**H6a**: Entrepreneurial experience positively moderates the mediating effect of entrepreneurial opportunity identification on the relationship between entrepreneurial course and independent entrepreneurship.

**H6b**: Entrepreneurial experience positively moderates the mediating effect of entrepreneurial opportunity identification on the relationship between entrepreneurial practice training and independent entrepreneurship.

**FIGURE 1:**
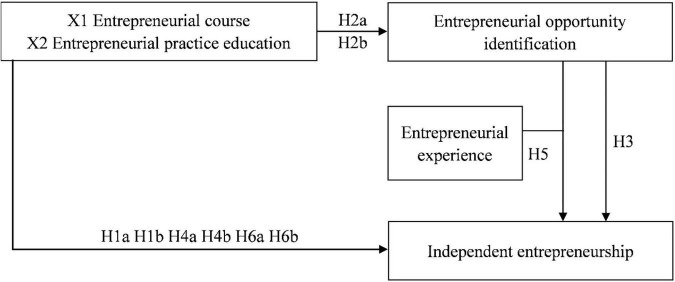
Theoretical model.

The theoretical model and calculation formula used in this study were exhibited as Figure 1.


(1) $$Y=c_{0}+c_{11}\,X_{1}+c_{12}\,X_{2}+e_{1}$$

(2) $$W=a_{0}+a_{11}\,X_{1}+a_{12}\,X_{2}+e_{2}$$

(3) $$Y=c_{0}^{\prime }+c_{11}^{\prime }\,X_{1}+c_{12}^{\prime }\,X_{2}+b_{1}\,M+e_{3}$$

(4) $$Y=b_{0}+b\,W+e_{4}$$

(5) $$Y=b_{0}+c^{\prime }\,W+b_{1}^{\prime }\,M+b_{2}^{\prime }\,W\,M+e_{5}$$

(6) $$Y=c_{0}^{\prime \prime }+c_{11}^{\prime \prime }\,X_{1}+c_{12}^{\prime \prime }\,X_{2}+c_{2}^{\prime }\,W+b_{2}\,M+b_{3}\,W\,M+e_{6}$$

## Materials and Methods

### Sampling

The sample used in this study was obtained by the China Institute of Innovation and Entrepreneurship Education through online surveys and offline interviews for 6,424 graduates with a postgraduation period not more than 1 year of graduation. An online invitation with the quick response (QR) code was sent out to the 5,000 graduates provided by the collaborating universities, outlining the brief purpose of the study. Corresponding data could be accumulated directly. The offline interview has followed the same pattern and the same questionnaire, and the interviewees were recruited by the administrative staff on campus during alumni gatherings. A total of 1,424 pieces of written questionnaires were recovered. The questions were elaborately generated, combining the coverage of core variables and the convenience of understanding and answering.

The data collection was performed within 31 provinces across China between 2017 and 2018. The output was shown as follows. For gender, 45.3% were men and 54.7% were women. For entrepreneurial experience, 34.4% of the respondents had an experience of establishing a start-up during their studentship period. They recognized its positive inspiration. Notably, 16.3% of the respondents had declared their willingness to start their own business, as the most favorable postgraduation choice; 10.7% of sampled graduates were from the provincial capital cities or province-level municipalities; 24.2% of the surveyed sample confirmed that their parents or other immediate family members have experienced entrepreneurship; and 27.0% of samples specialized in engineering and 13.1% graduated from “Double First-class” top universities. Refer Tables 1, 2.

**TABLE 1:** Distribution of majors.

Major	Quantity	Major	Quantity	Major	Quantity
Philosophy	66 (1.03%)	History	45 (0.70%)	Military	7 (0.11%)
Economics	888 (13.82%)	Science	579 (9.01%)	Management	1254 (19.52%)
Law	150 (2.33%)	Engineering	1734 (26.99%)	Art	272 (4.23%)
Pedagogy	355 (5.53%)	Agronomy	141 (2.19%)		
Literature	312 (4.86%)	Medicine	621 (9.67%)	Total	6424

**TABLE 2:** Distribution of demographics.

Eastern	Quantity	Central	Quantity	Western	Quantity
Beijing	80 (1.25%)	Shanxi	125 (1.95%)	Sichuan	529 (8.23%)
Tianjin	327 (5.09%)	Inner Mongolia	9 (0.14%)	Guizhou	55 (0.86%)
Hebei	216 (3.36%)	Jilin	30 (0.47%)	Yunnan	60 (0.93%)
Liaoning	360 (5.60%)	Heilongjiang	200 (3.11%)	Tibet	2 (0.03%)
Shanghai	125 (1.95%)	Anhui	195 (3.04%)	Shaanxi	373 (5.81%)
Jiangsu	179 (2.79%)	Jiangxi	195 (3.04%)	Gansu	42 (0.65%)
Zhejiang	622 (9.68%)	Henan	667 (10.38%)	Qinghai	34 (0.53%)
Fujian	91 (1.42%)	Hubei	73 (1.14%)	Ningxia	0 (0.00%)
Shandong	1418 (22.07%)	Hunan	54 (0.84%)	Xinjiang	8 (0.12%)
Guangdong	111 (1.73%)	Guangxi	54 (0.84%)		
Hainan	86 (1.34%)	Chongqing	104 (1.62%)		
Eastern Toal	3615 (56.27%)	Central Total	1706 (26.56%)	Western Total	1103 (17.17%)

### Measurement of Variables

On the basis of a well-established scale among existing studies, combined with the results of in-depth semi-structured interviews of scholars in the field of entrepreneurship education, the questionnaire for this study was designed after three rounds of modification and testing. The variables used in the research and analyses were derived from elements that have been recognized in the Pedagogy and widely applied. These variables were constructed based on the fundamentals in the mainstream field of interest to ensure validity, attempting to offer a panorama for the study. The content has covered entrepreneurial spirit, entrepreneurship theory-based course, and entrepreneurship practice training. Except for certain demographic characteristics and options requiring special clarification, the scales used in this study were in Likert 5-score measurement. Score 1 represented strongly disagree, while Score 5 was strongly agree. All the data were statistically processed using SPSS.

Entrepreneurship theory-based course questions were designed with reference to certain publications (Hosseini and Pouratashi, 2011; Wiley and Berry, 2015). Three measurement items were included, which were “diversified types of entrepreneurship education courses,” “closely integrated with major,” and “closely integrated era trends.”

Entrepreneurship practice training questions were designed with reference to certain publications (Greefs, 1998; Dos-Santos and Spann, 2011; Zou and Zhao, 2014). Five measurement items were included, which were “exclusive funding provided,” “integrated entrepreneurial practice services provided,” “independent entrepreneurship park provided,” “exclusive off-campus practice base provided,” and “practice projects highly integrated with professional learning.”

Entrepreneurial opportunity identification question was designed with reference to the 2017 GEM report “U: whether the respondent thinks there are good opportunities for starting a business in their local area.” Entrepreneurial opportunity identification was indicated by “good entrepreneurial opportunities in the local province.”

Virtual variables were used to measure entrepreneurial experience. For the question “among all the entrepreneurial practice activities you have participated in during school, which has helped you more,” digit 1 was marked for response “start a company outside school,” and the rest was recorded as 0.

Virtual variables were also used to measure independent entrepreneurship. For the question “the most favorable postgraduation choice,” digit 1 was marked for response “independent entrepreneurship,” and the rest was recorded as 0.

In terms of variable control, referring to the relevant research on college student independent entrepreneurship, four variables were selected, including gender, family entrepreneurship experience, major type (limited to engineering), and university type (limited to “Double First-class” top universities).

### Test for Reliability and Validity

The overall results derived from the test for reliability and validity were described as α = 0.921 and Kaiser-Meyer-Olkin (KMO) = 0.945, indicating that the overall scale has demonstrated decent reliability and validity. The value of α was larger than 0.7, leading to the fact that the reliability test and validity test results of each factor (refer to Table 3 for details) have all passed the internal consistency test, indicating that the reliability of each factor scale was satisfying. The KMO measure and Bartlett sphere test showed that all variables pass the test (KMO > 0.5), which met the factor analysis standard. The results of exploratory factor analysis showed that the factor load after item rotation was greater than 0.6, the combined reliability (CR) of all factors was greater than 0.7, and the average variance extracted (AVE) value of all factors was greater than 0.5. Each of the abovementioned three standards has clarified that the scale designed then utilized in the study had outstanding convergence validity.

**TABLE 3:** The reliability and validity of each factor.

Factor	Item	Factor load	*r* ^2^	KMO	α	CR	AVE
Entrepreneurial course	Diversified types	0.811	83.59%	0.738	0.902	0.904	0.759
	Integration with major	0.893					
	Integration with era trends	0.907					
Entrepreneurial practice training	Exclusive entrepreneurship park	0.860	82.89%	0.910	0.948	0.949	0.787
	Exclusive off-campus practice base	0.900					
	Integration with professional knowledge	0.905					
	Exclusive funding	0.854					
	Entrepreneurial practice services provided	0.915					

### Descriptive Statistics and Correlation Analysis

Each variable was paired to the rest of the variables for the calculation of variance dilation factor (VIF). Every VIF was less than 5, representing that multicollinearity did not happen. The results are shown in Table 4. Entrepreneurship theory-based course, entrepreneurship practice training, entrepreneurial opportunity identification, and entrepreneurship policy were positively correlated with independent entrepreneurship.

**TABLE 4:** Statistical parameters for each variable.

Variables	A	B	C	D	E	F	G	H	I
A	1								
B	−0.133**	1							
C	0.124**	−0.034**	1						
D	−0.045**	−0.316**	−0.078**	1					
E	−0.095**	−0.101**	−0.069**	0.235**	1				
F	0.089**	−0.042**	0.108**	–0.007	−0.056**	1			
G	0.071**	–0.021	0.082**	0.024	–0.002	0.818**	1		
H	0.120**	–0.022	0.145**	−0.060**	−0.104**	0.471**	0.433**	1	
I	0.060**	−0.114**	0.062**	0.009	–0.005	0.054**	0.047**	0.053**	1
Mean	0.160	1.550	0.240	0.270	0.130	3.555	3.688	3.180	0.340
Std Dev	0.370	0.498	0.428	0.444	0.337	0.925	0.906	0.994	0.475

**p < 0.01.

*A, Independent entrepreneurship. B, Gender. C, Family entrepreneurship experience. D, Major type (Engineering). E, University type (Double First-class). F, Entrepreneurship theory-based course. G, Entrepreneurship practice training. H, Entrepreneurial opportunity identification. I, Entrepreneurial experience.*

Considering the insufficiency of Harman’s single factor test, confirmatory factor analysis was performed to exclude the homology bias. The results are shown in Table 5. Among them, the single factor latent variable model with each item had a poor fitting, which indicated that common variance deviation was not detected among the variables used in this study. By comparing the fitting degree of the five-factor model and the four-factor model, it was found that the five-factor model used in this study was better than the four-factor model. In addition, the common method variance (CMV) was tested by setting nonmeasurable potential method factors. The output was found that the fitting statistics of the model did not appear obvious optimization, which further showed that the homologous method problem of the data used in this study was controlled, and the fitting degree of the model was satisfying.

**TABLE 5:** Validation factor analysis.

Model	*c*^2^/DF	CFI	GFI	AGFI	RMSEA	IFI	NFI
Single-factor	80.416	0.934	0.896	0.844	0.111	0.934	0.934
Four-factor	84.314	0.936	0.898	0.836	0.114	0.936	0.935
Five-factor	21.837	0.986	0.977	0.959	0.057	0.986	0.985
CMV	23.887	0.986	0.978	0.955	0.060	0.986	0.986

## Empirical Analysis

Considering that the dependent variable selection of independent entrepreneurship was a binary variable, which could not be tested by structural equation model and the plug-in called process, this study conducted multiple hierarchical regression analysis on the sampled data to verify the hypotheses. Among them, Models 1–6 were logistic regression with independent entrepreneurship as a dependent variable, and Models 7 and 8 were linear regression with entrepreneurial opportunity identification as a dependent variable.

**TABLE 6:** Parameters for main effect test.

Independent variable	Dependent variable	β	Result
X1 Entrepreneurship theory-based course	Y Independent entrepreneurship	0.141	**H1a** corroborated
X2 Entrepreneurship practice training	Y Independent entrepreneurship	0.053	**H1b** falsified

**TABLE 7:** Parameters for mediating effect test.

Independent variable	Dependent variable	β	Result
X1 Entrepreneurship theory-based course	Y Independent entrepreneurship	0.073	**H2a H4a** corroborated
X1 Entrepreneurship theory-based course	M Entrepreneurial opportunity identification	0.323	
M Entrepreneurial opportunity identification	X1 Entrepreneurship theory-based course Y Independent entrepreneurship	0.217	
X2 Entrepreneurial practice training	M Entrepreneurial opportunity identification	0.160	**H2b** corroborated **H4b** falsified

**TABLE 8:** Parameters for moderated effect test.

Independent variable	Dependent variable	β	Result
M Entrepreneurial opportunity identification	Y Independent entrepreneurship	0.259	**H3** corroborated
M Entrepreneurial opportunity identification	W Entrepreneurial experience	0.250	**H5** corroborated

**TABLE 9:** Parameters for moderated mediating effect test.

Independent variable	Dependent variable	β	Result
X1 Entrepreneurial course	Y Independent entrepreneurship	0.070	**H6a** corroborated
M Entrepreneurial opportunity identification	X1 Entrepreneurial course Y Independent entrepreneurship	0.151	**H6b** falsified
M Entrepreneurial opportunity identification	W Entrepreneurial experience	0.247	

**TABLE 10:** Multilevel regression analysis.

Variable	Y Independent entrepreneurship						M Entrepreneurship opportunity identification	
	Model 1	Model 2	Model 3	Model 4	Model 5	Model 6	Model 7	Model 8
Gender	−0.873** (0.073)	−0.875** (0.073)	−0.847** (0.073)	−0.852** (0.073)	−0.870** (0.074)	−0.827** (0.074)	−0.079** (0.026)	−0.048* (0.023)
Family entrepreneurship experience	0.620** (0.074)	0.576** (0.074)	0.537** (0.075)	0.545** (0.075)	0.533** (0.075)	0.526** (0.075)	0.312** (0.029)	0.203** (0.025)
Major (Engineering)	−0.418** (0.087)	−0.419** (0.087)	−0.404** (0.087)	−0.402** (0.087)	−0.401** (0.087)	−0.403** (0.087)	−0.091** (0.030)	−0.102** (0.026)
University Type (Double First-class)	−0.964** (0.141)	−0.964** (0.141)	−0.899** (0.141)	−0.902** (0.141)	−0.901** (0.141)	−0.899** (0.142)	−0.263** (0.037)	−0.210** (0.033)
X1 Entrepreneurial Course		0.141* (0.063)	0.073 (0.065)			0.070 (0.065)		0.323** (0.019)
X2 Entrepreneurship Practice training		0.053 (0.063)	0.014 (0.064)			0.013 (0.064)		0.160** (0.019)
M Entrepreneurship Opportunity Identification			0.217** (0.041)	0.259** (0.036)	0.190** (0.047)	0.151** (0.050)		
W Entrepreneurial experience					−0.334 (0.258)	−0.332 (0.257)		
M × W					0.250* (0.120)	0.247* (0.120)		
Hosmer-Lemeshow test	0.346	0.813	0.385	0.650	0.110	0.690		
R^2^							0.032	0.246
F							53.137	349.269

*p < 0.05, **p < 0.01.

### Main Effect Test

The regression coefficient of *X1 Entrepreneurship theory-based course* and *Y Independent entrepreneurship* was significantly positive (β = 0.141, *p* < 0.05). The effect of *X2 Entrepreneurship practice training* on *Y Independent entrepreneurship* was not significant (β = 0.053, *p* > 0.05). Derived from such results, hypothesis **H1a** was corroborated, and **H1b** was not supported. Refer Table 6.

### Mediating Effect Test

By substituting each index into formula (1), formula (2), and formula (3), the analysis results of Model 2, Model 3, and Model 7 were obtained. The direct effect of *X1 Entrepreneurship theory-based course* on *Y Independent entrepreneurship* was not significant (β = 0.073, *p* > 0.05). *X1 Entrepreneurial course* had a significantly positive impact on *M Entrepreneurial opportunity identification* (β = 0.323, *p* < 0.01). *M Entrepreneurial opportunity identification* had a significantly mediating effect on the relationship between *X1 Entrepreneurial course* and *Y Independent entrepreneurship* (β = 0.217, *p* < 0.01). Consequently, both hypotheses **H2a** and **H4a** were corroborated. As hypothesis **H1b** was not supported, it was impossible to test the mediating effect through the step-by-step method. However, *X2 Entrepreneurial practice training* had a significantly positive impact on *M Entrepreneurial opportunity identification* (β = 0.160, *p* < 0.01). It was revealed that hypothesis **H2b** was corroborated, while **H4b** was falsified. Refer Table 7.

To ensure the accuracy of the test, according to the study by Zhao et al. (2010), the product of coefficients was directly tested by bootstrap analysis with 1,000 samples (Felsenstein, 1985). The results showed that the interaction between *X1 Entrepreneurial course* and *M Entrepreneurial opportunity identification* was significant (95% CI = 0.035–0.077); thus, hypothesis **H4a** was supported.

### Moderated Effect Test

By substituting each index into formula (4), the analysis results of Model 4 showed that *M Entrepreneurial opportunity identification* had a significantly positive impact on *Y Independent entrepreneurship* (β = 0.259, *p* < 0.01). Thus, hypothesis **H3** was supported. By substituting each index into formula (5), the results of Model 5 demonstrated that the interaction between *M Entrepreneurial opportunity identification* and *W Entrepreneurial experience* had a significantly positive regression (β = 0.250, *p* < 0.05). The hypothesis **H5** was corroborated. Refer Table 8.

### Moderated Mediating Effect Test

According to the analysis results of Models 2 and 8, each index was substituted into formula (6) to get the analysis results of Model 5. Under the moderation of *W Entrepreneurial experience*, the direct effect of the *X1 Entrepreneurial course* on *Y Independent entrepreneurship* was not significant (β = 0.070, *p* > 0.05). *M Entrepreneurial opportunity identification* had a significantly mediating effect on the relationship between *X1 Entrepreneurial course* and *Y Independent entrepreneurship* (β = 0.151, *p* < 0.01). The interaction between *M Entrepreneurial opportunity identification* and *W Entrepreneurial experience* had significantly positive regression (β = 0.247, *p* < 0.05). Through the stepwise test, there was a moderating mediating effect emerged, and it showed positive regulation, namely, hypothesis **H6a** was supported. Hypothesis **H1b**, which should be a prerequisite for testing the moderating mediating effect using the stepwise method, was falsified. It is naturally transpired that **H6b** was also falsified.

To ensure the accuracy of the test, a 1,000-sample bootstrap analysis was conducted on the coefficient product of each variable with moderated mediating effect. It was found that the interaction items of *X1 Entrepreneurship theory-based course*, *M Entrepreneurship opportunity identification*, and *W Entrepreneurship experience* were significant (95% CI = 0.030–0.096). Hypothesis **H6a** was firmly corroborated. Refer Table 9.

## Discussion

The analysis results are shown in Table 10. According to the analysis of hypothesis **H1a**, entrepreneurship theory-based course had a significantly positive effect on independent entrepreneurship. After years of development, the content and form of entrepreneurship education in China are currently mature and perfect along with the socioeconomic rapid alteration. It has been derived from the lectures, case analyses, and other classic teaching approaches to the modern teaching modules including entrepreneurship plan design, case analysis contest, entrepreneurship project consultation, and workshops closely following the frontier of social focus (Charney and Libecap, 2000). Rich teaching content and diversified teaching forms have helped students to acquire more entrepreneurial knowledge. In terms of teaching forms, 50.08% of the respondents scored 3–5 points for “diversified types of courses.” The diversity of teaching methods has been recognized. Notably, 69.35% of the respondents had received case teaching and thought it was effective, 18.47% had attended exclusive lectures, 45.47% had participated in group discussion, 73.29% had carried out simulation practice, and 7.95% had studied online courses. In terms of teaching content, 53.50% of them scored 3–5 points for “entrepreneurship theory-based course content is closely combined with era trends.” Therefore, due to the increasingly diversified teaching methods and contents, different groups of students could find suitable learning methods and contents catering to their own interests and research directions, so as to obtain the knowledge and skills needed for independent entrepreneurship.

Entrepreneurship practice training had no significantly positive effect on independent entrepreneurship, which falsified hypothesis **H1b**. It is a highly counter-intuitive result as it has been widely believed that practice puts forward entrepreneurial activities. In this survey, 78.80% of the respondents scored 1–3 on “You think you have got enough knowledge, skills, and experience to start a business,” which, to a certain extent, reflected that those students, which have received entrepreneurial practice training realized their existing knowledge accumulation and ability training, were insufficient to tackle the risks and difficulties in the entrepreneurial process, leading to more cautious considerations in choosing independent entrepreneurship. Such a result intimated that college students may not choose to be independent entrepreneurship after participating in entrepreneurial practice. It revealed an unprecedented finding that happened after graduation.

According to the analysis of hypothesis **H2a**, entrepreneurship theory-based course had a significantly positive impact on the cultivation of entrepreneurial opportunity identification. In this survey, 20.00% of the respondents thought that the entrepreneurship theory-based course received on campus was the most helpful to improve their entrepreneurial ability, which showed the importance of entrepreneurship theory-based course to cultivate their entrepreneurial ability to a certain extent. Notably, 47.56% of the respondents scored 3–5 points in “entrepreneurship curriculum content is closely combined with major,” indicating that students recognized the rationality of teaching content within the curriculum. Entrepreneurship education content based on professional knowledge can be handier to assist students by essentially combining professional knowledge and entrepreneurship knowledge and then further cultivating the ability to identify entrepreneurial opportunities.

According to the analysis of hypothesis **H2b**, entrepreneurial practice training had a significantly positive impact on entrepreneurial opportunity identification ability. The university provided students with entrepreneurship contests (43.24% participation), practice in the campus entrepreneurship park (57.22% participation), entrepreneurship simulation training camp (34.34% participation), and enterprise management internship (44.75% participation). In addition, 53.50% of the respondents confirmed that entrepreneurship practice during studentship was most helpful to improve their entrepreneurial opportunity identification ability.

According to the analysis results of hypotheses **H1a**, **H2a,** and **H4a**, the total effect of entrepreneurship theory-based course on independent entrepreneurship was significant, the mediating effect of entrepreneurial opportunity identification was also significant, and the 95% CI of the interaction between entrepreneurship theory-based course and entrepreneurial opportunity identification did not contain 0. The mediating effect test and bootstrap analysis confirmed that entrepreneurial opportunity identification played a mediating role between entrepreneurial course and independent entrepreneurship. In addition, as the direct effect of entrepreneurship theory-based course on independent entrepreneurship was not significant, the mediating effect was exhibited as a full mediating effect. It represented that those entrepreneurial opportunities would be completely identified by entrepreneurship theory-based course, which indirectly affected the choice of independent entrepreneurship. This provided practical support for the current academic view that entrepreneurship education indirectly promoted students to start their own businesses by cultivating their entrepreneurial ability (Fayolle et al., 2006). According to the analysis results of hypotheses **H1b**, **H2b,** and **H4b**, the total effect of entrepreneurial practice training on independent entrepreneurship was not significant, and the mediating role of entrepreneurial opportunity identification between entrepreneurial practice training and independent entrepreneurship could not be verified through the mediating effect test. Therefore, entrepreneurial opportunity identification could not play a mediating role between entrepreneurial practice training and independent entrepreneurship.

According to the analysis results of hypothesis **H3**, entrepreneurial opportunity identification had a positive impact on guiding college students to start their own businesses. In addition, according to the analysis results of hypothesis **H5**, the interaction between entrepreneurial opportunity identification and entrepreneurial experience had a significantly positive effect on independent entrepreneurship. Entrepreneurial experience played a positive moderating role between entrepreneurial opportunity identification and the choice of independent entrepreneurship. Specifically, college students with entrepreneurial experience have accumulated entrepreneurial experience, and their entrepreneurial opportunity identification ability could be improved significantly after learning entrepreneurial knowledge and skills. They were more inclined to carry out independent entrepreneurship.

According to the analysis results of hypotheses **H1a**, **H2a,** and **H6a**, the mediating effect of entrepreneurial opportunity identification was still significant after adding entrepreneurial experience as a moderating variable. The 95% CI of the interaction among entrepreneurial course, entrepreneurial opportunity identification, and entrepreneurial experience did not contain 0, which meant that the mediating effect test and bootstrap analysis confirmed a moderated mediating effect model with the entrepreneurial course as the antecedent variable, entrepreneurial opportunity identification as the mediating variable, entrepreneurial experience as the moderating variable, and independent entrepreneurship as the dependent variable. In addition, as the direct effect of entrepreneurship theory-based course on independent entrepreneurship was not significant, the mediating effect was demonstrated as the full mediating effect between the entrepreneurship theory-based course and the choice of independent entrepreneurship.

## Conclusion and Implications

This study sampled fresh graduates who have received entrepreneurship education, through the construction of a moderated mediating model with entrepreneurship education as the antecedent variable. The role of entrepreneurship education in promoting college students to carry out independent entrepreneurship was analyzed, and the impact mechanism of entrepreneurial experience between entrepreneurial opportunity identification and independent entrepreneurship was verified. This study has raised and then tested several hypotheses. Hypotheses **H1a**, **H2a**, **H2b**, **H3**, **H4a**, **H5a**, **H5b,** and **H6a** were supported, while hypotheses **H1b**, **H4b,** and **H6b** were rejected. The result summary of hypothesis testing is shown in Table 11.

**TABLE 11:** Summary of study hypothesis test results.

Hypothesis	Content	Conclusion
**H1a**	Entrepreneurship theory-based course positively related to independent entrepreneurship	Corroborated
**H1b**	Entrepreneurship practice training positively related to independent entrepreneurship	Falsified
**H2a**	Entrepreneurship theory-based course positively related to entrepreneurial opportunities identification	Corroborated
**H2b**	Entrepreneurship practice training positively related to entrepreneurial opportunities identification	Corroborated
**H3**	Entrepreneurship opportunity identification positively related to independent entrepreneurship	Corroborated
**H4a**	Entrepreneurial opportunity identification plays a mediating role between entrepreneurial course and independent entrepreneurship	Corroborated
**H4b**	Entrepreneurial opportunity identification plays a mediating role between entrepreneurial practice training and independent entrepreneurship	Falsified
**H5**	Entrepreneurial experience plays a positive moderating role between entrepreneurial opportunity identification and independent entrepreneurship	Corroborated
**H6a**	Entrepreneurial experience positively moderates the mediating effect of entrepreneurial opportunity identification on the relationship between entrepreneurial course and independent entrepreneurship	Corroborated
**H6b**	Entrepreneurial experience positively moderates the mediating effect of entrepreneurial opportunity identification on the relationship between entrepreneurial practice training and independent entrepreneurship	Falsified

### Theoretical Implications

This study devoted three theoretical contributions to the mechanism of entrepreneurship education on college student independent entrepreneurship.

This research expanded new ideas of entrepreneurship education research. Previous studies on entrepreneurship education had shown that both entrepreneurship theory-based course and entrepreneurship practice training had exposed a positive role in promoting independent entrepreneurship. However, based on the data analysis of the questionnaire survey, this study confirmed that entrepreneurship theory-based course and entrepreneurship practice training had different impact directions. Entrepreneurship theory-based course had a significantly positive impact on independent entrepreneurship, while entrepreneurship practice training had not. But both entrepreneurship theory-based course and entrepreneurship practice training could generate a positive impact on the entrepreneurial opportunity identification. Therefore, this new research discovery expanded new ideas for the research of entrepreneurship education.

This research demonstrates that entrepreneurship education played an important role in entrepreneurial opportunity identification and indirectly promoted independent entrepreneurship. Previous studies on entrepreneurship education had majorly focused on the direct promotion of entrepreneurship education for college students (Fayolle et al., 2006). However, based on the data analysis of sampled graduates, this study finds that entrepreneurial opportunity identification played a complete mediating role between entrepreneurship curriculum education and independent entrepreneurship. Entrepreneurship theory-based course indirectly affects independent entrepreneurship through entrepreneurial opportunity identification, rather than directly promoting college students to choose independent entrepreneurship.

This research enriches the horizon on the process mechanism of entrepreneurship education affecting independent entrepreneurship. Previous studies on the mechanism of entrepreneurship education on independent entrepreneurship were mostly based on the mediating role of opportunity identification (Nidal and Norashidah, 2017) or the moderating role of entrepreneurial experience (Shirokova et al., 2015), but studies in the field of mediating mechanism were severely inadequate (Wei et al., 2019). Through investigation and exploration, this study analyzes the correlation between entrepreneurship education, entrepreneurial opportunity identification, entrepreneurial experience, and independent entrepreneurship in different dimensions and demonstrated the existence of moderated mediating effect model with entrepreneurship theory-based course as the antecedent variable, entrepreneurial opportunity identification as the intermediary variable, entrepreneurial experience as the moderating variable, and independent entrepreneurship as the dependent variable, which enriched the affecting mechanism of entrepreneurship education on independent entrepreneurship.

### Practical Implications

This study puts forward three practical implications for the mechanism of entrepreneurship education on independent entrepreneurship.

The teaching approaches of entrepreneurship theory-based course can be improved. By deepening the existing diversified entrepreneurship theory-based courses such as case analysis, exclusive lecture, group discussion, and simulation practice, online teaching of entrepreneurship theory-based course should be appropriately promoted to help different types of students find their own teaching content and obtain the relevant knowledge needed for entrepreneurship, so as to help them carry out independent entrepreneurship.

The form of entrepreneurship practice training should be expanded. On the basis of improving the existing entrepreneurial contest, on-campus entrepreneurial park practice, entrepreneurial simulation training camp, and other practice activities within the campus, off-campus practice activities should be appropriately developed, enterprise management internship, for instance. Enriched forms of practical education provided conditions for college students to carry out practical activities and help them test whether it could be plausible for entrepreneurship at the minimum cost.

The support system for college students to start their own businesses should be improved. Cooperation between the university and local government/enterprise should be further strengthened to build a set of support systems, in terms of venues, funds, personnel, and policies, capable for the student to establish off-campus start-ups, as a method of willingness promotion.

### Limitations and Prospects

Due to limitations such as funds, time, and other factors, the proportion of samples collected in this study was not satisfactorily abundant. Since this is a cross-sectional design study, the sampled data represented only the situation of college students who have received entrepreneurship education within 1 year after graduation instead of a more common period of 3–5 years. Although the variables used in the research and analyses were dedicated generated within the field of Pedagogy, they could not completely avoid the deviation caused by individual subjective opinions. Besides, there were no controlled variables, which possibly led to the deviation even when three rounds of test modification had been carried out.

In the future, this study can further discover the relationship between variables at different time points or periods. Top priority should be given to the in-depth comparative analysis in the follow-up studies. The collected data can be further compared with general university graduates in China to conduct a comparative analysis of data grouping and subspecialty. Moreover, diverse datasets to reexamine and multiexamine the relationship of the factors on the various background graduates can further be utilized to justify the entrepreneurial opportunity results. It is also practicable to explore the impact of other personal characteristics or situational factors as moderators on independent entrepreneurship, in order to find more moderators that can affect the relationship between entrepreneurship education, entrepreneurial opportunity identification, and independent entrepreneurship. It is worthy of considering absorbing other entrepreneurial capabilities that are not involved in this study and then exploring whether entrepreneurial self-efficacy, entrepreneurial awareness, and other mediating variables are applicable in the model constructed.

## Data Availability Statement

The raw data supporting the conclusions of this article will be made available by the authors, without undue reservation.

## Ethics Statement

Ethical review and approval was not required for the study on human participants in accordance with the local legislation and institutional requirements. The patients/participants provided their written informed consent to participate in this study.

## Author Contributions

All authors listed have made a substantial, direct, and intellectual contribution to this work, and approved it for publication.

## Conflict of Interest

The authors declare that the research was conducted in the absence of any commercial or financial relationships that could be construed as a potential conflict of interest.

## Publisher’s Note

All claims expressed in this article are solely those of the authors and do not necessarily represent those of their affiliated organizations, or those of the publisher, the editors and the reviewers. Any product that may be evaluated in this article, or claim that may be made by its manufacturer, is not guaranteed or endorsed by the publisher.
